# A Humanized Anti-VEGF Rabbit Monoclonal Antibody Inhibits Angiogenesis and Blocks Tumor Growth in Xenograft Models

**DOI:** 10.1371/journal.pone.0009072

**Published:** 2010-02-05

**Authors:** Yanlan Yu, Pierre Lee, Yaohuang Ke, Yongke Zhang, Qiu Yu, Jonathan Lee, Mingzhen Li, Jialiang Song, Jungang Chen, Jihong Dai, Fernando Jose Rebelo Do Couto, Zhiqiang An, Weimin Zhu, Guo-Liang Yu

**Affiliations:** 1 Department of Urology, Sir Run-Run Shaw Hospital, College of Medicine, Zhejiang University, Hangzhou, People's Republic of China; 2 Epitomics, Inc., Burlingame, California, United States of America; 3 Hangzhou Yikang Biotech, Inc., Hangzhou, People's Republic of China; Hong Kong University, Hong Kong

## Abstract

Rabbit antibodies have been widely used in research and diagnostics due to their high antigen specificity and affinity. Though these properties are also highly desirable for therapeutic applications, rabbit antibodies have remained untapped for human disease therapy. To evaluate the therapeutic potential of rabbit monoclonal antibodies (RabMAbs), we generated a panel of neutralizing RabMAbs against human vascular endothelial growth factor-A (VEGF). These neutralizing RabMAbs are specific to VEGF and do not cross-react to other members of the VEGF protein family. Guided by sequence and lineage analysis of a panel of neutralizing RabMAbs, we humanized the lead candidate by substituting non-critical residues with human residues within both the frameworks and the CDR regions. We showed that the humanized RabMAb retained its parental biological properties and showed potent inhibition of the growth of H460 lung carcinoma and A673 rhabdomyosarcoma xenografts in mice. These studies provide proof of principle for the feasibility of developing humanized RabMAbs as therapeutics.

## Introduction

Antibodies are becoming a major drug modality due to the high specificity and affinity to their targets [1]. More than two dozen therapeutic monoclonal antibodies are currently approved for the treatment of cancer and other human diseases [1], [2]. Most therapeutic antibodies developed to date were either chimeric or humanized murine antibodies due to early availability of the mouse hybridoma technology [1], [3]. Recently, transgenic mice and *in vitro* phage display were employed to generate fully human therapeutic antibodies [4], [5], [6], [7]. These antibody platforms have their own limitations; it is desirable to have access to other antibody sources to increase the overall success rate of developing more effective antibody therapies [8], [9]. Rabbit has a robust immune system to generate antibodies with high affinity and specificity [10], [11], [12], [13], but routine generation of rabbit monoclonal antibodies (RabMAbs) only become possible recently due to the availability of a stable rabbit hybridoma fusion partner cell line 240E-W2 [14], [15]. To evaluate rabbit antibodies for therapeutic use, we generated a panel of neutralizing RabMAbs against human vascular endothelial growth factor-A (VEGF), an angiogenic growth factor [16], [17]. Overexpression of VEGF correlates with advanced tumor stage or tumor invasiveness in various types of human cancers and blockage of VEGF/VEGFR-2 signaling is a clinically proven strategy for the treatment of a number of cancers [18], [19]. We humanized a rabbit monoclonal antibody with high affinity and specificity to VEGF using a unique strategy known as mutational lineage guided (MLG) humanization [20], [21]. Humanization of RabMAbs is an essential step in developing therapeutic antibodies so as to minimize the potential human anti-rabbit antibody response when administrated in humans [22]. We demonstrate that the humanized anti-VEGF antibody retains the parental affinities and biological activities. We also showed that neutralizing VEGF signaling by the humanized RabMAb blocks tumor growth and inhibits angiogenesis in two *in vivo* models. The results described in this report demonstrate the potential for developing humanized RabMAbs as therapeutics.

## Results

### Generation of Anti-Human VEGF RabMAbs

Six rabbits were immunized with full length human VEGF fused to Fc domain of rabbit IgG recombinantly expressed in mammalian cells. Two rabbits whose sera gave the best neutralizing activities by the receptor-ligand binding assay were selected (data not shown). A total of 235 hybridomas with specific binding to human VEGF were identified. Bevacizumab, a humanized mouse anti-human VEGF antibody approved for clinical treatment of colorectal cancer, and an anti-human Factor VIII antibody were used as positive and negative controls, respectively, in the VEGF/VEGFR-2 binding ELISA assay.

**Figure 1:**
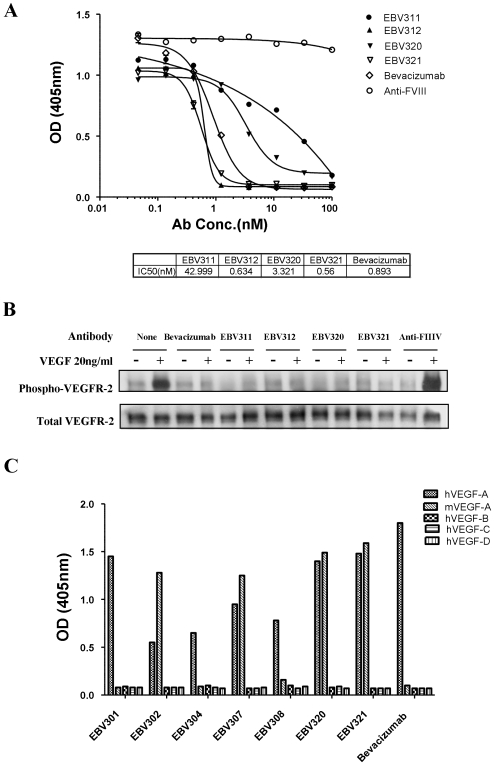
*In vitro* characterization of anti-VEGF RabMAbs. (**A**) Dose-dependent inhibition of VEGF/VEGFR-2 interaction by 4 representative anti-VEGF RabMAbs. Bevacizumab and a non-relevant anti-human Factor VIII RabMAb were included as controls. Points represent means of three replications and error bars represent standard deviations. The IC_50_ values are shown in the table below. (**B**) Inhibition of VEGF-stimulated receptor tyrosine phosphorylation in 293/KDR cells in the presence of neutralizing antibodies against VEGF. Four representative anti-VEGF RabMAbs inhibited VEGF stimulated VEGFR-2 phosphorylation in 293/KDR cells. Bevacizumab and a non-relevant anti-human Factor VIII RabMAb were used as positive and negative controls, respectively. Total VEGFR-2 staining was performed as sample quantitative controls. (**C**) Specificity and cross-reactivity of anti-VEGF RabMAbs. A panel of seven representative anti-human VEGF antibodies exhibited no reactivity to human VEGF-B, -C, -D. Four of the seven antibodies (EBV302, EBV307, EBV320 and EBV321) were cross-reactive with mouse VEGF.

A panel of 15 anti-VEGF hybridoma antibodies that block human VEGF binding to VEGFR-2 were identified from the 235 hybridomas with specific binding to human VEGF. The IC_50_ of the 15 VEGF neutralizing antibodies ranged from 0.4 to 43 nM when compared with Bevacizumab (IC_50_ = 0.9 nM) in the receptor-ligand binding assay (**Table 1**). Titration and IC_50_ results of four representative anti-VEGF RAbMAbs (EBV311, EBV312, EBV320, and EBV321) are shown in **Figure 1A**. We further evaluated the ability of these antibodies to block receptor tyrosine phosphorylation triggered by VEGF binding [23]. HEK293 cells expressing full length VEGFR-2 were stimulated with human VEGF in the presence or absence of VEGF neutralizing antibodies. **Figure 1B** shows that all four representative anti-VEGF RabMAbs block receptor phosphorylation upon VEGF stimulation.

**Table 1:** In vitro characterization of anti-VEGF RabMAbs.

Clone name	Inhibition of VEGF/VEGFR-2 interaction IC_50_ (nM)*			Mouse VEGF crossreactivity
EBV301	0.991	±	0.086	−
EBV302	25.158	±	1.018	+
EBV303	1.023	±	0.079	+
EBV304	3.420	±	0.589	−
EBV305	5.182	±	0.556	−
EBV307	2.615	±	0.081	+
EBV308	1.835	±	0.081	−
EBV310	0.559	±	0.012	+
EBV311	42.999	±	6.210	+
EBV312	0.634	±	0.060	+
EBV313	1.243	±	0.040	+
EBV320	3.321	±	0.055	+
EBV321	0.560	±	0.026	+
EBV322	0.410	±	0.006	+
EBV361	1.818	±	0.361	+
Bevacizumab	0.893	±	0.044	−

* Mean ± standard deviation of three replications.

All 15 VEGF neutralizing antibodies are highly specific to human VEGF-A and they do not bind to human VEGF-B, -C, and -D. Results for 7 representative antibodies are shown in **Figure 1C**. To test cross-reactivity of the neutralizing antibodies, mouse VEGF was included in the assay. Four of the 15 antibodies bind to the human VEGF only and 11 of the antibodies are human/mouse cross-reactive (**Table 1** and **Figure. 1C**). As expected, Bevacizumab is human VEGF specific.

### Sequence and Structure Analysis of the VEGF Neutralizing RabMAbs

**Figure 2:**
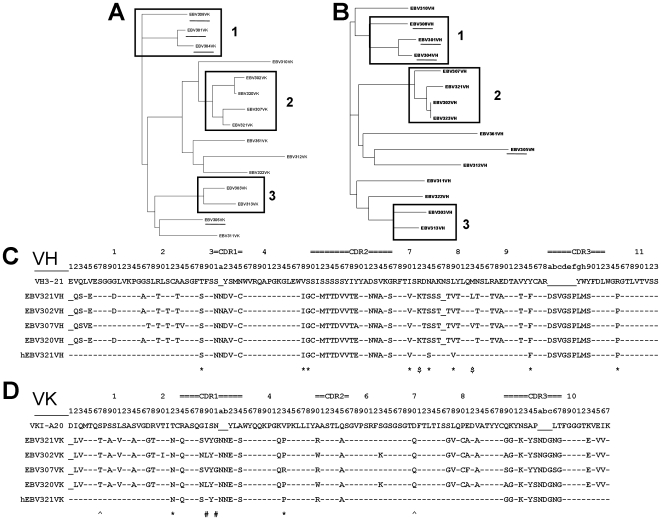
Mutational lineage guided humanization of anti-VEGF RabMAb EBV321. (**A–B**) Phylogenetic analysis of VK (**A**) and VH (**B**) amino acid sequences of 15 neutralizing anti-VEGF RabMAbs by Clustal X. The human antigen-specific clones are underlined. RabMAbs of the same lineage group are boxed and labeled as 1, 2 or 3. (**C–D**) Alignments of VH (**C**) and VK (**D**) protein sequence of the EBV321 lineage group 2 (EBV302, EBV307, EBV320 and EBV321) with human germline and humanized EBV321 sequences. ‘–’ denotes residues that are identical at the corresponding positions. ‘*’ denotes the residues in RabMAb framework regions potentially involved in CDR contacts or inter-chain contacts. ‘$’ denotes the residues considered not critical to the structural activity. ‘#’ denotes the residues humanized in the CDR region. Chothia numbering scheme and Kabat CDR loop definition were used [55], [56].

Phylogenetic trees based on variable region amino acid sequences of light (VK) and heavy (VH) chains of the 15 VEGF neutralizing RabMAbs are shown in **Figure 2A, B**. Three of the 4 human VEGF specific antibodies (EBV301, EBV304, and EBV308) are clustered in both the heavy and light chain lineages (group 1 in **Figure 2A, B**). Similarly, six of the 11 cross-reactive antibodies (EBV302, EBV307, EBV320, and EBV321 in group 2; EBV303 and EBV313 in group 3) are also clustered in both the heavy and light chain lineages (**Figure 2A, B**). The other cross-reactive antibodies (EBV310, EBV311, EBV312, EBV322 and EBV361) are dispersed within the large cluster where groups 2 and 3 are located in the light chain tree (**Figure 2A**). In the heavy chain lineage tree, two cross-reactive antibody (EBV312 and EBV361) is clustered with group 2 and another two (EBV311 and EBV322) are clustered with group 3 (**Figure 2B**). Of the 30 sequences in the heavy and light chain lineage trees, there are only two outliers. The human VEGF specific antibody EBV305 is grouped with the cross-reactive antibodies (**Figure 2A, B**). The heavy chain of cross-reactive antibody EBV310 is clustered with group 1 which contains three human VEGF specific antibodies (**Figure 2B**). To investigate the correlation between binding activity and phylogenetic relationship, heavy and light chains of the 15 antibodies were paired to generate 225 antibodies and their binding activities to VEGF were determined (**Table 2**). Strong structural activity relationship was observed. For example, the heavy chain of clone EBV321 was able to pair with the light chains of clones in the same lineage such as clones EBV302, EBV307 and EBV320 (Group 2 in **Figure 2A, B**), but when paired with the light chains of clones in different lineages it was not able to reconstitute antigen binding activity (**Table 2**). Similarly, light chain of clone EBV321 could pair with heavy chains from clones in the same lineage group, EBV302, EBV307 and EBV 320, but not with heavy chains from different lineage groups (**Table 2**). The same was for antibodies in groups 1 and 3 (**Figure 2A, B** and **Table 2**). Two exceptions were the heavy chains of EBV311 and EBV322 which were able to pair with a number of light chains in different lineage groups. However, their light chains could only pair with their corresponding heavy chains (**Table 2**). Based on *in vitro* biological activity, antigen binding affinity, and sequence analysis, we selected EBV321 for humanization.

**Table 2:** Antigen binding activity of random pairs of 15 anti-VEGF antibody H and L chains.

	301L	302L	303L	304L	305L	307L	308L	310L	311L	312L	313L	320L	321L	322L	361L
**301H**	**++**	**–**	**–**	**++**	**–**	**–**	**++**	**–**	**–**	**–**	**–**	**–**	**–**	**–**	**–**
**302H**	**–**	**++**	**–**	**–**	**–**	**++**	**–**	**–**	**–**	**–**	**–**	**++**	**++**	**–**	**–**
**303H**	**–**	**–**	**++**	**–**	**–**	**–**	**–**	**–**	**–**	**–**	**++**	**–**	**–**	**–**	**–**
**304H**	**++**	**–**	**–**	**++**	**–**	**–**	**++**	**–**	**–**	**–**	**–**	**–**	**–**	**–**	**–**
**305H**	**–**	**–**	**–**	**–**	**++**	**–**	**–**	**–**	**–**	**–**	**–**	**–**	**–**	**–**	**–**
**307H**	**–**	**++**	**–**	**–**	**–**	**++**	**–**	**++**	**–**	**–**	**–**	**++**	**++**	**–**	**–**
**308H**	**++**	**–**	**–**	**++**	**–**	**–**	**++**	**–**	**–**	**–**	**–**	**–**	**–**	**–**	**–**
**310H**	**–**	**–**	**–**	**–**	**–**	**++**	**–**	**++**	**–**	**–**	**–**	**–**	**+**	**–**	**–**
**311H**	**++**	**++**	**–**	**++**	**–**	**++**	**–**	**++**	**++**	**–**	**–**	**++**	**++**	**–**	**–**
**312H**	**–**	**–**	**–**	**–**	**–**	**–**	**–**	**–**	**–**	**++**	**–**	**–**	**–**	**–**	**–**
**313H**	**–**	**–**	**++**	**–**	**–**	**–**	**–**	**–**	**–**	**–**	**++**	**–**	**–**	**–**	**–**
**320H**	**–**	**++**	**–**	**–**	**–**	**++**	**–**	**–**	**–**	**–**	**–**	**++**	**++**	**–**	**–**
**321H**	**–**	**++**	**–**	**–**	**–**	**++**	**–**	**–**	**–**	**+**	**–**	**++**	**++**	**–**	**–**
**322H**	**–**	**–**	**++**	**–**	**–**	**–**	**–**	**+**	**–**	**–**	**++**	**–**	**–**	**++**	**–**
**361H**	**–**	**–**	**–**	**–**	**–**	**–**	**–**	**–**	**–**	**–**	**–**	**–**	**–**	**–**	**++**

The data were obtained from antigen binding ELISA with the antigen coated on a 96-well plate. ‘–’, antigen binding signal <3X of background reading; ‘+’, antigen binding signal between 3**–**10× of background reading; ‘++’, antigen binding signal >10× of background reading.

### Humanization by Mutational Lineage Guided (MLG) Antibody Engineering

The MLG method was used to humanize EBV321 into a human IgG1 subclass with kappa light chain. First, the heavy (VH) and light chain (VK) variable region sequences of EBV321 were blasted against the human germline VH and VK database [24]. The closest human germline sequences, VH3-21/JH2 and VKI-A20/JK4 were identified as the template for EBV321 humanization (**Figure 2C, D**). Second, antibody sequences (EBV302, EBV307, and EBV320) within the EBV321 lineage (group 2) were aligned (**Figure 2A, B, C, D**). In the EBV321 lineage, the four clones have an identical VH CDR3, share similar sequences in other CDRs, and contain the same number of amino acid residues in the variable region (**Figure 2C, D**). Third, the rabbit residues in the framework regions potentially involved in CDR contacts or inter-chain contacts were identified based on the knowledge from human and mouse antibodies. These residues, marked by “*”, were not changed (28S, 48I, 49G, 70V, 74S, 79V,95F, 105P in VH and 22N, 43P in VK) (**Figure 2C, D**) [25]. At CDR1 position 29 of VK, V is interchangeable to L within the group and given that amino acids I and L are structurally related, the original residue V in clone EBV321 was changed to the human germline residue I at position 29 as marked by “#” (**Figure 2D**). Similarly, at CDR1 position 31 of VK, G is interchangeable to N within the group, so it was mutated to the human germline residue N as marked by “#” (**Figure 2D**). Residues considered not critical to the structural activity of the antibody as based on the phylogenetic analysis were humanized as marked by “$” (72K→R and 83L→M of VH) (**Figure 2C**). Residues at position 7T and 70Q of VK were substituted with human germline residues S and D, respectively, as marked by “ ^” because they are facing solvent. In addition, the N-terminal residues of VK (N'-LV→N'-DIQ) and VH (N'-QS→N'-EVQ) were changed to match the human germline sequences. The parental EBV321 antibodies before humanization were 69% and 75% identical, respectively, to the human germline VH and VK frameworks. After MLG engineering, the frameworks of the final humanized EBV321 (hEBV321) are 92% and 97% identical, respectively, to the human germline VH and VK frameworks.

### Epitope Mapping

**Figure 3:**
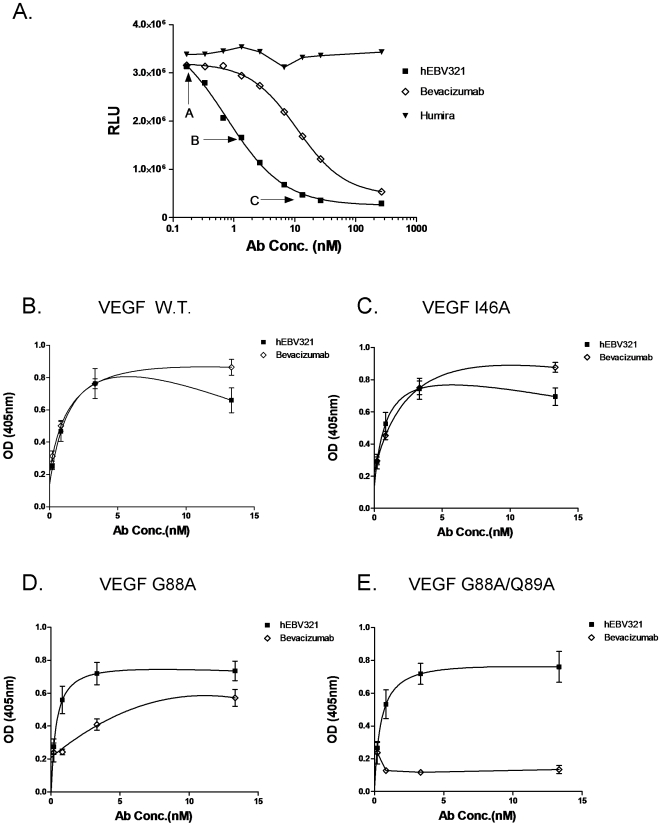
Comparison of hEBV321 and Bevacizumab binding properties to human VEGF. A. VEGF competition ELISA measuring the binding of hEBV321 to VEGF coated on ELISA plate in the presence of increasing concentration of competitor (hEBV321, Bevacizumab or an irrelevant antibody Humira). B-E. Direct binding of hEBV321 and Bevacizumab to various forms of human VEGF captured on ELISA plate. B. VEGF wild type; C. VEGF I46A; D. VEGF G89A; E. VEGF G88A/Q89A.

A direct competition ELISA against human VEGF was performed to determine whether the VEGF-A165 binding epitope of hEBV321 is similar to that of Bevacizumab. The parental rabbit EBV321 was used as a reference antibody. The binding of EBV321 to VEGF was assessed in the presence of increasing amount of hEBV321 or Bevacizumab competitor. Binding of EBV321 to VEGF-A165 was not affected by hEBV321, Bevacizumab or the negative control antibody Humira at low concentrations (arrow A, **Figure 3A**). At 5 fold excess, hEBV321 inhibited the binding of EBV321 by 50% while Bevacizumab showed very little competition (arrow B, **Figure 3A**). Further increase in competitor concentration continued to reduce EBV321 binding and the binding of EBV321 was reduced to about 10% and 37% by hEBV321 and Bevacizumab, respectively (arrow C, **Figure 3A**). At intermediate concentration range, Bevacizumab showed only partial competition with EBV321 as compared to hEBV321 (**Figure 3A**), however EBV321 binding to VEGF was abolished by either competitor at the highest concentration tested (1000 fold excess). The control antibody Humira showed no competition even at maximum concentration.

To further confirm that the epitope recognized by hEBV321 is different from that of Bevacizumab, binding of hEBV321 to mutated forms of VEGF A165 that affect Bevacizumab binding were assessed. Binding of hEBV321 and Bevacizumab to the wild type VEGF and VEGF-I46A were similar (**Figures 3B and 3C**). Binding of hEBV321 to VEGF-G88A resembled its binding to the wild type VEGF, but binding of Bevacizumab to VEGF-G88A was substantially reduced as compared to hEBV321 (**Figure 3D**). hEBV321 retained binding to VEGF G88A/Q89A at levels comparable to wild type, but Bevacizumab binding was abolished, reflecting distinct epitope binding (**Figure 3E**).

### Biological Activities of the Humanized Anti-VEGF RabMAb

**Figure 4:**
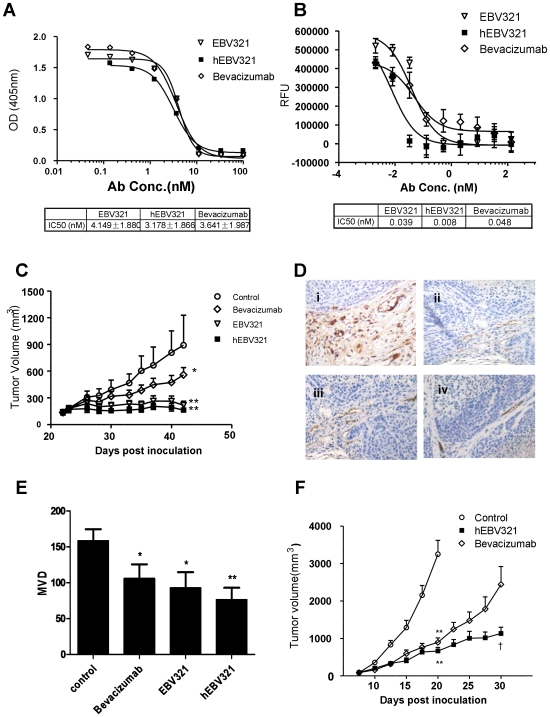
Humanized EBV321 retains full biological activities *in vitro* and *in vivo*. (**A**) Comparison of hEBV321 and its parental RabMAb EBV321 in the receptor-ligand interaction ELISA assay. Bevacizumab was included as a positive control. Points represent the means of three replicates and error bars represent standard deviations. The IC_50_ values are shown in the table below. (**B**) Dose-dependent inhibition of HUVEC proliferation by RabMAb EBV321, hEBV321, and Bevacizumab. Points represent the means of three replicates and error bars represent standard deviations. The IC_50_ values are shown in the table below. (**C**) Inhibition of tumor growth *in vivo* by anti-VEGF RabMAbs EBV321 and hEBV321 in NCI-H460 xenograft model. Bevacizumab, EBV321, and hEBV321 were dosed at 5 mg/kg 3 times per week for a total of 9 doses (8 mice per group). Error bars represent the standard deviation. (**D**) Photomicrographs of immunohistochemistry staining with an anti-CD34 mAb (x400). Representative areas of tumor sections from cohorts receiving saline as control (**i**), Bevacizumab (**ii**), EBV321 (**iii**) and hEBV321 (**iv**). (**E**) MVD scoring of CD34 staining in NCI-H460 xenograft tumors. (**F**) Inhibition of tumor growth by hEBV321 in A673 xenograft model. Animals were *i.p.* injected with normal saline, 5 mg/kg hEBV321 and Bevacizumab, 3 times per week for a total of 9 doses (8 to 11 mice per group). Symbols and bars, mean + standard deviation. Significant difference when compared to the control group by ANOVA was denoted with *: *P*<0.05 or **: *P*<0.01. †, significant difference compared with the bevacizumab group (*P*<0.05, student's *t* test).

To examine whether hEBV321 retains the properties of the parental antibody, we first measured the antigen binding affinities of hEBV321 and its parental EBV321 by surface plasmon resonance (SPR); Bevacizumab was included in the same assay. When VEGF was used as the analyte, the *K_D_*s of EBV321 and hEBV321 were 419 pM and 11.5 pM, respectively (**Table 3**). The *K_D_* for Bevacizumab was 16.6 nM (**Table 3**). Next, we compared the blocking activity of hEBV321 and its parental EBV321 in the ligand-receptor ELISA assay. hEBV321 blocked the binding of human VEGF to human VEGFR-2 extracellular domain with a similar dose-response curve and IC_50_ value as its parental EBV321 (4.1 nM and 3.2 nM for EBV321 and hEBV321, respectively) (**Figure 4A**). The ability of these two antibodies to inhibit VEGF-induced receptor phosphorylation was examined in a quantitative phospho-ELISA assay. The amount of tyrosine phosphorylated receptors decreased with increasing amount of hEBV321 and its parental RabMAb EBV321. We further determined the activity of hEBV321 and EBV321 in inhibiting VEGF-stimulated growth of HUVECs and results showed that both clones displayed dose-dependent inhibition on HUVEC growth (**Figure 4B**). RabMAb EBV321 exhibited similar potency as Bevacizumab (IC_50_ = 0.039 nM *vs.* 0.048 nM), whereas hEBV321 (IC_50_ = 0.008 nM) was about 5-fold and 6-fold more potent than its parental RabMAb EBV321 and Bevacizumab, respectively.

**Table 3:** Binding affinities of anti-VEGF-A RabMAb EBV321 and its humanized version.

	*k_on_* (1/Ms)	*k_off_* (1/s)	*K_D_* (M)	χ^2^
EBV321-VEGF	2.85×10^−6^	1.192×10^−3^	4.19×10^−10^	4.76
hEBV321-VEGF	1.63×10^−6^	1.88×10^−5^	1.15×10^−11^	1.88
Bevacizumab	3.84×10^−4^	5.78×10^−4^	1.66×10^−8^	0.90

*k_on_*, association rate constant; *k_off_*, dissociation rate constant; *K_D_*, equilibrium constant; χ^2^, chi-squared distribution.

### Efficacy of hEBV321 in the Human NCI-H460 Lung Carcinoma and A673 Rhabdomyosarcoma Mouse Xenograft Models

The *in vivo* efficacy of hEBV321 and its parental rabbit antibody EBV321 were evaluated in two mouse xenograft models of human cancers. In the first model, BALB/c nude mice were subcutaneously inoculated with NCI-H460 human lung cancer cells and treatment began when established tumors reached an average size of about 100 mm^3^. Antibodies were administered intraperitoneally at 5 mg/kg/dose, 3 times per week for a total of 9 doses. All three antibodies (hEBV321, EBV321 and Bevacizumab) showed significant inhibition of tumor growth as compared to saline control at dose of 5 mg/kg (hEBV321, *P* = 0.0004; EBV321, *P* = 0.0015; Bevacizumab, *P* = 0.042) (**Figure 4C**). After three weeks of treatment, tumors were excised and measured. Tumor growth inhibition [(C-T)/C%] by hEBV321 was 81.5% whereas the Bevacizumab was 37.2% (*P* = 0.003, hEBV321 vs. Bevacizumab). At a lower dose (2.5 mg/kg), effective tumor growth inhibition was also observed in hEBV321 treated mice (60% inhibition, *P* = 0.010), but not significant in the Bevacizumab group (11% inhibition, *P* = 0.567) (data not shown). Histological examination of NCI-H460 tumor xenograft sections was performed to determine the microvessel density. Tumor sections stained with an antibody against vascular endothelial marker CD34 revealed that treatment with 5 mg/kg of hEBV321, EBV321, and Bevacizumab resulted in a weaker CD34 staining and a lower density of CD34 positive blood vessels as compared to saline treated control (*P* = 0.0034, *P* = 0.020, and *P* = 0.040 *vs.* control for hEBV321, EBV321, and Bevacizumab, respectively) (**Figure 4D, E**).

In the A673 rhabdomyosarcoma xenograft model, antibodies were administered intraperitoneally at 5 mg/kg/dose, 3 times per week for a total of 9 doses. The animals were sacrificed when the tumors reached 3000 mm^3^ in size. The average tumor size in the control group exceeded 3000 mm^3^ after 5 doses (day 20 after inoculation). Both antibodies showed significant inhibition of tumor growth throughout the treatment (**Figure 4F**). The inhibition of tumor growth by hEBV321 and Bevacizumab was similar after 5 doses (79.6% and 72.5% respectively *vs.* control, *P*<0.001). However, at the end of 3 weeks, average tumor size in the hEBV321 and Bevacizumab treated groups were 1132 mm^3^ and 1814 mm^3^, respectively, and the difference between the two treatments was statistically significant (*P* = 0.019) (**Figure 4F**).

## Discussion

In this study we have demonstrated the generation of highly specific and potent neutralizing antibodies against human VEGF using the rabbit monoclonal technology platform (RabMAbs). In addition to their high affinity and specificity, RabMAbs exhibit several other advantages [8], [11], [12], [14], [26]. First, due to the size of the rabbit spleen, a larger pool of hybridomas can be generated from fusion of a single immunized rabbit spleen. Second, RabMAbs generally have higher affinity than mouse monoclonal antibodies [9]. Higher binding affinity is one of the critical parameters in selection of potent and efficacious therapeutic antibodies [27], [28], [29]. Third, antibodies from rabbits are likely cross-reacting with antigens from the mouse. In our anti-human VEGF neutralization antibody panel, more than 50% of RabMAbs, including EBV321, were cross-reactive to the mouse VEGF. For therapeutic antibody development, a cross-reactive antibody against the human and mouse targets provides a convenient way to evaluate the antibody in mouse-based *in vivo* efficacy models and eliminates the need for a surrogate antibody during preclinical and animal toxicity studies. The lack of cross-reactive antibodies has been an obstacle in preclinical development of therapeutic antibodies [30], [31]. In addition to cross-species reactivity, rabbit antibodies are believed to recognize a greater variety of epitopes than antibodies generated in mice [11], [32], but experimental data supporting this claim is lacking. The length of CDR3 in the EBV321 light chain is 12 residues (**Figure 2C, D**) which is longer than the average length of light chain CDR3 in human or mouse IgGs [33]. The longer light chain CDR3 may explain the higher diversity of rabbit antibody repertoires [34]. Finally, rabbit antibodies are more closely related to human than mouse-derived antibodies [26].

Using the *in vitro* functional assay results and sequence analysis data from a VEGF neutralizing RabMAb panel, we have humanized clone EBV321 by selectively replacing the non-human residues in both frameworks and a CDR (VK-CDR1). An interesting feature was observed from the phylogenetic analysis. The heavy chain phylogenetic tree overlaps with the light chain phylogenetic tree suggesting that the heavy and light chains from the same B-cell have co-evolved during B cell maturation. It further suggested that antibodies in a lineage group are derived from a single progenitor B cell. Such co-evolution of heavy and light chains has been previously reported in vertebrates [35], [36]. Further sequence analysis of antibodies within the same lineage showed that both the frameworks and the CDRs are conserved (**Figure2C, D**). The sequence variation among the antibodies in the same lineage group are likely the result of somatic mutations of the progenitor B cell. A lineage related antibody group defined by sequence analysis was further confirmed by their cross reactivity to human and murine antigens, and exchangeability of heavy and light chains among the antibodies. While some of the mutations contribute to antigen binding and structural stability, others are not critical. For example, at position 72 of VH, R and K are acceptable in lineage group 2 (**Figure 2C**); therefore, we humanized the K residue in EBV321 to R. Another example is position 31 of VK (CDR1) (**Figure 2D**) at which G and N are interchangeable and we humanized the G residue in EBV321 to N. Only one humanized version was tested and it retained the *in vivo* and *in vitro* properties of the parental antibody, suggesting that humanization guided by both biological and sequence information allows for retention of full antibody activity. CDR grafting or resurfacing based humanization methods rely primarily on sequence information and by these methods it is difficult to predict the role a specific residue plays in antibody activity [37], [38]. As a result, multiple humanized versions are often tested in labor intensive *in vitro* and *in vivo* assays [38], [39]. Furthermore, unlike other protocols, the MLG method allows for humanization of both the framework and CDR regions.

hEBV321 demonstrated potent tumor inhibition in a NCI-H460 human xenograft model. Although hEBV321 exerted a more prominent inhibition on tumor growth and angiogenesis than Bevacizumab when administered at the same dosage and schedule, the higher potency of hEBV321 is likely in part due to its recognition of the mouse VEGF. It has been reported that mouse VEGF binds to human VEGFR-2 ECD *in vitro* [40]. Mouse VEGF can also stimulate human HUVAC cell proliferation [41]. It has also been reported that the existence of host-derived VEGF might cause residual growth or drug resistance of human tumor xenografts in nude mice treated with antibodies against human VEGF [31], [42]. In a comparative study, Bevacizumab is about 90% effective at inhibiting HM-7 and A673 xenograft growths in nude mice, whereas its effectiveness drops below 50% in HPAC tumors. This may be due to a higher level of host-derived VEGF surrounding the tumor mass in the HPAC model than in the other two models, and the fact that Bevacizumab only reacts with the tumor-derived human VEGF [31]. The extent of contribution of stroma-derived VEGF to NCI-H460 tumor growth has not been reported. Therefore, in order to compare the *in vivo* efficacy of blocking human tumor-derived VEGF, we further compared hEBV321 and Bevacizumab in an A673 rhabdomyosarcoma xenograft model, in which stromal VEGF is nearly non-existent [31]. As was seen in the NCI-H460 model, hEBV321 showed increased efficacy as compared to Bevacizumab in the A673 xenograft model, indicating that inhibition of stromal VEGF is not necessary for the increased effectiveness of hEBV321.

Antagonists of VEGF signaling have been developed for anti-angiogenic therapy, however, side effects have been observed clinically, as is the case for Bevacizumab [43], [44]. *In vivo* study showed that hEBV321 inhibits tumor growth and angiogenesis effectively at a dose as low as 0.25 mg/kg (data not shown). If this translates into the clinical setting, hEBV321 may reduce the unwanted side-effects associated with high dosage [45], [46]. The mechanism for higher potency of hEBV321 in the preclinical models is not clear, but at least three factors may contribute: higher affinity, cross-reactivity to the mouse target, and recognition of a different epitope. The affinity of hEBV321 is about 1444 fold higher than Bevacizumab to VEGF (11.5 pM for hEBV321 vs. 16.6 nM for Bevacizumab) (**Table 3**). The reported binding affinity of Bevacizumab to VEGF was 2 nM [31]. The discrepancy between the reported 2 nM and our 16.6 nM Kd value for Bevacizumab may be explained by the different formats used in the two experiments. Liang et al. coupled the VEGF to the surface and used Bevacizumab as the analyte [31]. The 2 nM Kd was a potential measurement of avidity rather than affinity since IgG is bivalent.

VEGF blocking ELISA yielded similar IC_50_ values for EBV321 and hEBV321 (in the range of 3 to 4 nM; **Figure 4A**), whereas a 40 fold difference in SPR Kd values was observed for the two antibodies (11.9 and 419 pM for hEBV321 and EBV321, respectively; **Table 3**). This can be explained by the fundamental difference between the two methods. While SPR such as Biacore measures true Kd, solution IC_50_ determination by ELISA does not measure true affinity constants for a given binding pair [47]. Based on the definition of Kd, close to 100% of the binding partners exist in the bound form once both partners are at a concentration 5–10 fold in excess of the Kd. Therefore, under the ELISA conditions in which the antibody concentrations are in the IC_50_ range of 3–4 nM, which represents ∼100 fold and ∼10 fold of Kd for hEBV321 (11.9 pM) and EBV321 (419 pM) respectively, all the antibodies exist in the bound form, thus binding in this assay is more of a stoichiometry driven event, rather then an affinity driven event.

The binding site on human VEGF to Bevacizumab and VEGFR-2 have been examined previously [48], [49], [50]. Competitive binding and mutagenesis studies suggest that hEBV321 and Bevacizumab have partially overlapping epitopes, but with different amino acid binding requirements. This is also supported by the fact that hEBV321 cross-reacts to mouse VEGF while Bevacizumab does not. The Bevacizumab VEGF binding profiles are consistent with the previous report that VEGF residues G88 and G89, but not I46 are important binding determinants for Bevacizumab [49], [51]. The competitive ELISA results showed that hEBV321competes for VEGF binding with the parental EBV321 in a dose responsive manner, suggesting that the humanization process did not alter the antigen binding epitope.

In summary, we have identified a panel of neutralizing RabMAbs against human VEGF with high affinity and specificity. These RabMAbs specifically block VEGF/VEGFR-2 interaction and VEGF-induced receptor phosphorylation. In addition to binding the human target, many of them can also recognize the mouse ortholog. We have humanized one RabMAb and demonstrated efficacy of the humanized antibody hEBV321 in neutralizing VEGF functions both *in vitro* and *in vivo*. These results suggest that rabbit monoclonal antibody technology combined with the MLG humanization process represent a novel platform for efficiently generating high quality candidates for therapeutic antibody development. hEBV321 is currently in preclinical development.

## Materials and Methods

### Cell Lines and Proteins

Human umbilical vein endothelial cells (HUVECs) from Lonza (Walkersville, MD) were maintained in Endothelial Basal Medium-2 (EGM®-2, Lonza) supplemented with 2% fetal bovine serum and growth factors (BulletKit®, Lonza). Cell line 293/KDR which stably expresses human VEGFR-2 was purchased from SibTech, Inc (Brookfield, CT). Other cell lines used in this report were purchased from the American Type Culture Collection (Manassas, VA). NCI-H460, 293/KDR, and A673 cells were maintained in DMEM medium supplemented with 10% fetal bovine serum at 37°C in 5% CO_2_. The rabbit IgG Fc-hVEGF (human VEGF_165_) and Fc-VEGFR-2 fusion protein expression constructs were prepared by cloning human VEGF or VEGFR-2 extracellular domain DNA fragments at the *Bgl*II and *Bam*HI site C-terminal to the rabbit IgG Fc in the mammalian expression plasmid vector pTT5 (National Research Council, Canada). The recombinant fusion proteins were expressed in transient transfected HEK293-6E cells and purified from cell culture supernatant by Protein A column chromatography. Recombinant human VEGF_165_ was from Shanghai Primegene Bio-Tech Co. (Shanghai, China) and R&D System (Mineapolis, MN). Other recombinant human VEGF proteins (VEGF-B, VEGF-C, and VEGF-D) and mouse VEGF were from R&D System.

### Generation of Anti-Human VEGF RabMAbs

New Zealand white rabbits were immunized subcutaneously with 0.2 mg recombinant Fc-hVEGF in TiterMax™ Gold Adjuvant (Sigma-Aldrich Corp., St. Louis, MO). After the initial immunization, animals were boosted 3 times in a 3 week-interval. Rabbits with the highest sera titers were intravenously boosted with 0.4 mg immunogen four days before splenoectomy. Hybridoma fusion was performed according to established protocol with minor modifications [14]. Briefly, splenocytes were harvested from the immunized rabbit and fused with rabbit plasmacytoma cells 240E-W2 [15] using PEG4000 (Sigma Chemical, St. Louis, MO) and selected by HAT (hypoxanthine, aminopterin, and thymidine). At end of the selection, hybridoma clones growing in the original 96-well plates were transferred to new 96-well plates with a medium change. Hybridoma supernatants were collected and screened for antigen binding and neutralization of VEGF/VEGFR-2 interaction. Hybridomas that were positive in the binding and neutralization assays were subcloned, expanded and frozen for future use.

### Cloning and Expression of Recombinant RabMAbs

mRNA was isolated from hybridoma cells and reverse-transcribed to cDNA using the Qiagen TurboCapture mRNA kits (Qiagen, Inc, Vaencia, CA). DNA fragments for the entire L chain and the variable region of H chain (VH) of rabbit IgG were amplified by PCR with rabbit H and L chain primers. The L chain fragment was cloned into the pTT5 mammalian expression vector and the VH fragment was fused in-frame to the constant region of H chain in another pTT5 vector. For each hybridoma clone, three plasmid DNA clones for H and L chains were sequenced and expressed as recombinant RabMAbs for validation of cloning and subsequent characterization. To express recombinant RabMAbs, the L and H chain plasmids were co-transfected into 293-6E cells (National Research Council, Canada) and supernatants were harvested 5 days after transfection. The recombinant RabMAbs in the supernatants were purified through protein A column, dialyzed in PBS buffer and quantified at OD280 nm. For animal studies, the transiently expressed recombinant RabMAbs were purified through Protein A, HiTrap Phenyl HP and Hiprep 26/10 column (GE Healthcare Bio-Sciences, Piscataway, NJ), quantified and filled into vials under sterile condition. All data present in this paper were using recombinant RabMAbs, unless it is specified otherwise.

### VEGF/VEGFR-2 Interaction ELISA

A total of 5 µg/ml Fc-VEGFR-2 (ECD) fusion protein was coated on ELISA plates at 4°C overnight and blocked in 1% BSA in TBS containing 0.05% Tween-20 for 1 hour at room temperature. Anti-VEGF RabMAbs, Bevacizumab (Roche, Basilea, Switzerland) or control IgG were pre-incubated with recombinant human VEGF (0.5 µg/ml final concentration) for 1 hour before transferring to the VEGFR-2-coated ELISA plates. After 1 hour of incubation, the plates were washed with TBST twice and VEGF bound to immobilized VEGFR-2 was detected by a mouse anti-VEGF_165_ monoclonal antibody (Sigma-Aldrich Corp., St. Louis, MO), followed by the addition of goat anti-mouse IgG alkaline phosphatase conjugated antibody (Fisher Scientific/Pierce Biotechnology, Rockford, IL). ELISA plates were developed with p-nitrophenyl phosphate substrate and absorbance at 405 nm was recorded. All experiments were performed in triplicates.

### Endothelial Cell Proliferation Assay

HUVECs were seeded at a density of 4,000 cells/well in 96-well plates. A total of 50 µl of the tested antibodies at indicated concentrations were pre-incubated with 50 µl VEGF (15 ng/ml) for 1 hour before added to plates and incubated in EGM®-2 without growth factor (assay medium) at 37°C, 5% CO_2_ for 72 hours. Then 10% AlamarBlue® (Serotec, Oxford, UK) was added to each well and incubated for an additional 24 hours. Cell viability was measured by a Wallac Victor V multilabel HTS counter (PerkinElmer, Waltham, MA) with an excitation wavelength of 530 nm and emission wavelength of 590 nm. All studies were conducted twice and in triplicates for each sample concentration.

### Affinity Measurement of Anti-VEGF RabMAbs

Affinities were measured by BIAcore 3000 (BIAcore, Inc., Uppsala, Sweden). Antibodies were immobilized on CM5-Sepharose chips coated by standard amine coupling chemistry to a density of about 600 response units (RU) [52]. Measurements were carried out at 25°C by injecting 250 µl of recombinant VEGF165 (R&D Systems) at concentrations from 0.2 to 50 nM with a flow rate of 35 µl/min. The dissociation duration for bound analytes was 15 minutes in HBS-P running buffer (10 mM HEPES, pH 7.4, 150 mm NaCl and 0.005% surfactant P20, filtered and degassed). Data were analyzed with the BIAevaluation software (version 3.2RC1) by applying a 1∶1 binding model. The obtained sensorgrams were fitted globally over the whole range of injected concentrations and simultaneously over the association and dissociation phases. Equilibrium dissociation constants were then calculated from the rate constants (*K_D_*  =  *k_off_/k_on_*).

### Inhibition of VEGF Stimulated VEGFR-2 Phosphorylation

The 293/KDR cells (1×10^6^ cells/well) were starved in DMEM containing 0.2% fetal bovine serum for 4 hours. Anti-VEGF RabMAbs were pre-mixed with 20 ng/ml human VEGF for 1 hour at room temperature. The antibody/antigen mixtures were then added to the 293/KDR cells and incubated for 3 minutes at 37°C. After washing with cold PBS, the cells were lysed with cold lysis buffer containing 20 mM Tris-HCl (pH 7.5), 150 mM NaCl, 1 mM EDTA, 1% Triton X-100, 0.5 mM sodium orthovanadate, 1 mM phenylmethylsulfonyl fluoride, and complete protease inhibitor mixture on ice (Cell Signaling, Danvers, MA). The crude cell lysates were clarified by centrifugation and protein concentration was determined by Bicinchoninic Acid Kit (Sigma-Aldrich Corp., St. Louis, MO). The phosphorylation of VEGFR-2 on tyrosine residues were assessed by Western immunoblotting and ECL detection using a mouse anti-phosphotyrosine PY99 monoclonal antibody (Santa Cruz Biotechnology, Santa Cruz, CA) and rabbit anti-VEGFR-2 polyclonal antibody (Epitomics Inc., Burlingame, CA) as primary antibodies.

### Mutational Lineage Guided (MLG) Humanization of RabMAbs

A phylogenetic tree was built by alignment of VH and VL amino acid sequences of selected RabMAbs. Sequence alignment and phylogenetic analysis were performed using the ClustalX software [53]. The parental RabMAb and the most closely related human germline sequence were then aligned. Residues which are known not to be structurally critical and/or subjected to change during the *in vivo* maturation process were identified in the MLG analysis and humanized. DNA encoding humanized VK and VH were synthesized by MCLab (South San Francisco, CA, USA). The human IgG signal peptide and a Kozak sequence were engineered at the 5′ends of the VK and VH sequences. The humanized VK fragment was then cloned into human CK pTT5 vector at the *Hin*dIII and *Bsi*WI site. Similarly, humanized VH was cloned into a separate human IgG1 CH pTT5 vector at *Hin*dIII and *Nhe*I site. Expression, purification and quantification of the humanized RabMAbs are the same as those for recombinant RabMAbs.

### Direct Competion ELISA

ELISA microplate (Microlite 2, Thermo Scientific) was coated with human VEGF-A165 (Shanghai PrimeGene Bio-Tech Co., Ltd.) in carbonate-bicarbonate buffer at 4°C overnight. The plate was washed and blocked in 1%BSA TBST buffer. Bevacizumab, hEBV321, or negative control antibody Humira were mixed individually at increasing concentration with an equal volume of hEBV321 (0.04 µg/ml). The antibody mixtures were then transferred to the VEGF coated ELISA plate (0.05 µg/ml) and incubated for 1 hr at room temperature. After washing, bound hEBV321 was detected by peroxidase conjugated AffiniPure goat anti-rabbit IgG and Fc fragment specific secondary antibody (Jackson Immunoresearch, Cat. No. # 111-035-046). The ELISA plate was then processed for ECL substrate development (Femtoglow HRP Substrate Plus, Cat.# SHRPE21007, Michigan Diagnostic LLC) and the chemiluminesence signal (RLU) was recorded by Wallac Victor V (PerkinElmer, 1420 Multilabel HTS Counter).

### VEGF Mutants Cloning and Expression

Human VEGF-A 165 mutants I46A, G88A, and G88A/Q89A were generated using QuikChange XL Site-Directed Mutagenesis Kit (Stratagene, Catalog #200516) with human Fc-VEGF A 165 wild type DNA construct as the template according to the manufacturer's instructions. The mutagenic primers were synthesized by Elim Biopharmaceuticals, Inc. and the mutations are confirmed by DNA sequencing performed by MCLAB. The primers used are: I46A forward - gatgagatcgagtacgccttcaagccatcctgtgtg; I46A reverse - cacacaggatggcttgaaggcgtactcgatctcatc; G88A forward - caaacctcaccaagcccagcacataggagag; G88A reverse - ctctcctatgtgctgggcttggtgaggtttg; G99A/Q89A forward - cggatcaaacctcaccaagccgcccacataggagagatg; and G99A/Q89A reverse - catctctcctatgtgggcggcttggtgaggtttgatccg.

The wild type and mutant VEGF-A-Fc-fusion proteins were expressed by transient transfection of HEK-293E cells. Two independent clones for each mutant were expressed and analyzed. Briefly, HEK-293 cells were plated in 2 ml of freestyle 293 expression medium (Invitrogen, Cat. No. 12338) in 6-well tissue culture plates and transfected with various DNA complexed with 293fectin Transfectin Reagent in OptiMEM Complexing Medium (Invitrogen, Cat. Nos. 12347-019 and 31985-070). The culture medium was supplemented with TN1 peptone 24 hrs post-transfection and harvested on day 5. The culture media were centrifuged at 1200 g for 5 min. The cleared supernatants were used for VEGF capture ELISA binding experiment.

### VEGF Capture ELISA Binding Assay

ELISA microplates (Microlon, high binding, Greiner Bio-One, Cat. No. # 650061) were coated with AffiniPure goat anti-rabbit IgG (H+L) (Jackson ImmunoResearch, Cat. No. 111-005-003) in 0.05M carbonate-bicarbonate buffer at 4°C overnight. The plates were then blocked in 1% BSA in TBST for 2 hr at 37°C. Fc-VEGF protein supernatants were added to the well and incubated for 1 hr at 37°C. The plates were washed in TBST before addition of anti-VEGF antibodies (hEBV321 or Bevacizumab) at various concentrations for 1 hr at 37°C. This was followed by washing and incubation at 37°C with alkaline phosphatase conjugated goat anti-human IgG (H+L) secondary antibody (Pierce/Thermo Scientific, Cat. No. 31310). After washing in TBST, the ELISA plates were developed by addition of p-nitrophenyl phosphate solution at room temperature. The reaction was stopped by addition of NaOH and the plates were read at 405 nm with an ELISA plate reader (Labsystems Multiskan Ascent Photometric plate reader).

### Establishment and Treatment of Human Tumor Xenografts in BALB/c Nude Mice

NCI-H460 or A673 cells were maintained in DMEM/10%FBS medium until 80% confluence. Female BALB/c nu/nu mice 6–8 weeks of age were obtained from Shanghai SLAC Laboratory Animal Co. Ltd. (Shanghai, China). The mice were housed at five/cage in microisolator units under humidity- and temperature-controlled conditions with 12-hour light/dark cycle, and fed with a standard sterile laboratory diet (Zhejiang Chinese Medical University, Hangzhou, China). Xenografts were established by subcutaneous injection of 1×10^7^ cells/mouse into the dorsal flanks. When tumors reached an average volume of about 100 mm^3^ (50∼200 mm^3^), the animals were randomized into groups. Antibodies were administered intraperitoneally at 5 mg/kg per dose or as indicated. The control group received sterile saline. Dosing was administered 3 times per week for a total of 9 doses. Tumor size and body weight were recorded every 2 to 3 days. Perpendicular dimensions of the tumor were measured using a Vernier scale caliper. Tumor volumes were calculated according to the following equation: Volume  =  (width)^2^ × length/2. Animals were euthanized three weeks after the first treatment and tumors were removed and weighed. All procedures were conducted in accordance to Animal Care and Use Committee guidelines of Zhejiang Chinese Medical University.

To assess tumor histology and microvessel density (MVD), MVD scores of tumor xenografts were determined by quantifying immunohistochemical staining of the CD34 marker using a modified method described previously [54]. Briefly, tissue sections were processed, deparaffinized, rehydrated, and quenched for endogenous peroxidase activity. Murine vessels in tumor xenografts were immunostained with rabbit anti-mouse CD34 antibody (Zhongshan Goldenbridge Biotechology Co., LTD., Beijing, China) for 1.5 hours at 37°C. After washing with PBS three times, sections were incubated with goat anti-rabbit IgG-horseradish peroxidase for 30 minutes. The slides were developed with diaminobenzidine (DAB) and counterstained with Haematoxylin. The entire tumor section was scanned under a light microscope at low power magnification (×100) to identify “hot spots”, which are the areas of the highest neovascularization. Individual microvessels were then counted under a high power (×400) field to obtain a vessel count in a defined area, and the average vessel count in five hot spots was taken as the MVD.

### Statistical Analysis

Results are expressed as mean ± standard deviation, unless otherwise indicated. Data and graphs were analyzed with Graphpad Prism software (Graphpad Software Inc., La Jola, CA). Statistical significance of multiple comparisons was determined by ANOVA and difference between two groups was determined by two-tailed Student's *t* test. A *p*-value of <0.05 was taken as statistically significant.
